# Cloning and identification of novel hydrolase genes from a dairy cow rumen metagenomic library and characterization of a cellulase gene

**DOI:** 10.1186/1756-0500-5-566

**Published:** 2012-10-13

**Authors:** Xia Gong, Robert J Gruninger, Meng Qi, Lyn Paterson, Robert J Forster, Ron M Teather, Tim A McAllister

**Affiliations:** 1 Agriculture and Agri-Food Canada, Lethbridge Research Centre, Lethbridge, Alberta, T1J 4B1, Canada; 2 Feed Research Institute, Chinese Academy of Agricultural Sciences, Beijing, China

**Keywords:** Endoglucanase, Ruminal microorganisms, BAC library, Dairy cow

## Abstract

**Background:**

Interest in cellulose degrading enzymes has increased in recent years due to the expansion of the cellulosic biofuel industry. The rumen is a highly adapted environment for the degradation of cellulose and a promising source of enzymes for industrial use. To identify cellulase enzymes that may be of such use we have undertaken a functional metagenomic screen to identify cellulase enzymes from the bacterial community in the rumen of a grass-hay fed dairy cow.

**Results:**

Twenty five clones specifying cellulose activity were identified. Subcloning and sequence analysis of a subset of these hydrolase-positive clones identified 10 endoglucanase genes. Preliminary characterization of the encoded cellulases was carried out using crude extracts of each of the subclones. Zymogram analysis using carboxymethylcellulose as a substrate showed a single positive band for each subclone, confirming that only one functional cellulase gene was present in each. One cellulase gene, designated *Cel14b22*, was expressed at a high level in *Escherichia coli* and purified for further characterization. The purified recombinant enzyme showed optimal activity at pH 6.0 and 50°C. It was stable over a broad pH range, from pH 4.0 to 10.0. The activity was significantly enhanced by Mn^2+^ and dramatically reduced by Fe^3+^ or Cu^2+^. The enzyme hydrolyzed a wide range of beta-1,3-, and beta-1,4-linked polysaccharides, with varying activities. Activities toward microcrystalline cellulose and filter paper were relatively high, while the highest activity was toward Oat Gum.

**Conclusion:**

The present study shows that a functional metagenomic approach can be used to isolate previously uncharacterized cellulases from the rumen environment.

## Background

Cellulose is a principal component of plant cell walls. Efficient cellulose hydrolysis requires the synergistic activity of three classes of cellulase: endo-1,4-β-glucanase (EC3.2.1.4), cellobiohydrolase (EC3.2.1.91) and β-glucosidase (EC3.2.1.21). In recent years, interest in plant cell-wall-degrading enzymes, including cellulases, has increased due to the numerous potential industrial applications of these enzymes [1]. They have been widely applied in the textile and laundry, food and feed, pulp and paper, baking, waste treatment, and biomass-based alcohol production industries [2]. Growing concerns about the worldwide shortage of non-renewable fuel and rising oil prices as well as greenhouse gas emissions have led to an increasing interest in the development of alternative environmentally-friendly biofuels [3].

Cellulases are produced by a variety of organisms, including archaea, prokaryotes, fungi, plants, and animals. The most effective known natural systems for rapid biomass conversion, however, involve complex communities of microorganisms, primarily prokaryotes and fungi, maintained in a symbiotic relationship with an animal host [4]. This apparent need for complexity is a consequence of the biochemical intricacy of the biomass that serves as the fermentation substrate, a complex, physically and chemically linked mixture of cellulose, hemicellulose, xylan, waxes, and lignins.

Perhaps one of the best characterized examples of an effective complex biomass degrading community is that harboured within the rumen [4]. The natural diet of ruminants is mainly plant material, and most of the digestion of plant mass takes place under anaerobic conditions within the rumen. Many hydrolase genes have been isolated and characterized from cultivable ruminal microbes [5] however, it is generally accepted that a large proportion of the microorganisms in many complex natural environments remain uncultured [6,7]. Even for the relatively intensively studied rumen microbial community it is estimated that more than 85% of its members have still not been cultivated [5]. This estimate is based on the phylogenetic diversity of rumen microbial communities in deer, sheep and cattle as revealed by 16S/18S rDNA amplification sequencing techniques, denaturing gradient gel electrophoresis, terminal restriction fragment length polymorphism, and suppressive subtractive hybridization methods [8-12]. This unexplored microbial diversity represents an untapped source of potentially novel and unique enzymatic activities and metabolic pathways that can be applied to industrial biomass conversion [7,13,14].

Metagenomic approaches have been widely used to isolate novel biocatalysts from environmental samples [15]. Several metagenome derived hydrolase genes have been identified in metagenomic libraries prepared from various environmental samples, including those from the rumen [14,16-19]. The goals of this study were to clone and characterize novel cellulases from the rumen microbial community. To this end, a functional screen of a bacterial artificial chromosome (BAC) metagenomic library from the cow rumen was implemented to identify proteins involved in the degradation of polysaccharides by uncultivated rumen microorganisms.

## Results

### Metagenomic library construction and screening

A metagenomic BAC library of ~6000 clones was constructed with high molecular weight DNA isolated by a freeze grinding technique from dairy cow rumen samples. Screening for hydrolase activities resulted in the identification of ten independent clones expressing carboxymethyl cellulase (CMCase) activities, nine expressing β-glucosidase activities and seven expressing hydrolase activities for other substrates were isolated (Table 1).

**Table 1:** Putative glycosyl hydrolases obtained from subclones expressing cellulase activities from the cow rumen metagenome

**BAC clone**	**Subclone**	**First blastx match**	**Expect**	**Identity/Similarity(%)**
13-I03	p21	β-glucosidase (B. formatexigens DSM 14469, ZP_05346034)	2.00 × 10^-23^	37/51
13-N07	p296	Cel8B (F. succinogenes, ABU45499)	8.00 × 10^-176^	89/94
14-B22	p13	CH5 (Uncultured microorganism, AFO64636)	0	78/86
14-F03	p75	Cel9B (F. succinogenes, AAC44386)	0	71/83
3-M02	p28	Cel9B (F. succinogenes, AAC44386)	0	63/75
3-N18	p20	β-glucosidase (P. bergensis DSM 17361, ZP_06005092)	0	70/79
3-N18	p13	β-glucosidase (Bacteroides sp. 2_1_7, ZP_05286991)	2.00 × 10^-115^	50/66
3-P24	p44	Cel9B (F. succinogenes, AAC44386)	0	71/83
4-E05	p13	β-glucosidase (P. bergensis DSM 17361, ZP_06005092)	4 × 10^-29^	60/72
4-E05	p10	β-xylosidase (uncultured rumen bacterium,CAP07659)	1 × 10^-117^	60/71
5-C07	p44c	Cel5H (F. succinogenes, ABU45500)	7.00 × 10^-60^	39/56
5-I05	p20	β -glucosidase related glycosidases (Ruminococcus obeum A2-162, CBL22609)	9.00 × 10^-13^	31/50
5-K18	p52	β-glucosidase (B. formatexigens DSM 14469, ZP_05346034)	2.00 × 10^-36^	43/56
6-C02	p2	Cel5H (F. succinogenes, ABU45500)	0	53/69
6-I10	p35	Cel5H (F. succinogenes, ABU45500)	0	53/69
6-K13	p41	Cel5H (F. succinogenes, ABU45500)	7.00 × 10^-60^	39/56
6-L14	p61	Cel5H (F. succinogenes, ABU45500)	1.00 × 10^-5^	40/60
7-A06	p24	putative xylanase (Bacteroides fragilis 3_1_12, ZP_05281078)	2.00 × 10^-9^	29/45
7-D10	p19	β-glucosidase (P. bergensis DSM 17361, ZP_06005092)	0	65/75
7-L24	p1	β-glucosidase (P. bergensis DSM 17361, ZP_06005092)	4.00 × 10^-116^	62/73

The total library encompassed an estimated 900 Mb of insertion DNA. The positive rate of hydrolase activity in the library was approximately 0.15% of examined clones, or one expressed hydrolase gene per 10 Mb of insert DNA. Subcloning and sequence analysis of a subset of these hydrolase-positive clones identified twenty endoglucanase genes (Table 1). Predicted amino acid sequences generally indicated moderate to low homology and similarity (average 70%) to the cellulases in NCBI databases. Ten endoglucanases with CMCase activity were selected for further study. Glycoside hydrolase families 5, 8 and 9 were represented by six, one, and three of these ten endoglucanases, respectively (Table 1).

### Characterization of enzyme activities for the subcloned cellulases

**Figure 1:**
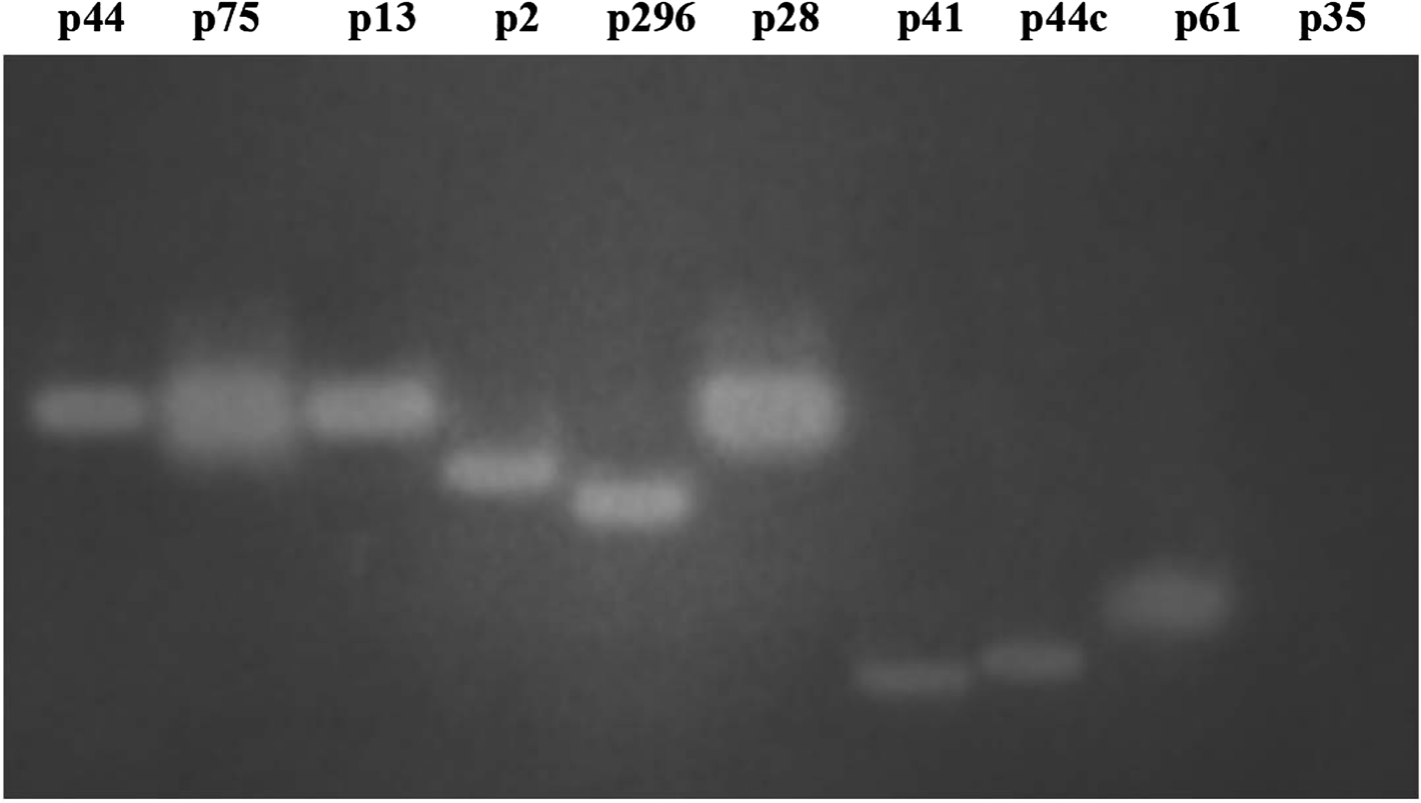
**Zymogram analysis of the 10 cloned cellulases in native polyacrylamide gel eletrophoresis.** CMCase activities were detected on CMC agar replica plates of the polycrylamide gel.

On the basis of a CMCase zymogram with native PAGE, each of the subclones selected for further study appeared to produce only a single active protein which was responsible for the observed activity, except for p35 where no positive band was observed (Figure 1). The CMCase activity of the 10 cellulases was assayed over a range of pH values and temperatures. The pH optima ranged from 6 to 7, while the optimal temperatures for all were between 40 to 50°C (Table 2). All the cellulases were also tested for their substrate specificities (Table 3). The CMCases showed a diverse range of activity with six showing maximal activity toward oat gum, one toward barley glucan, two toward CMC, and one toward lichenan. The activity of these enzymes towards methyl cellulose, filter paper, Avicel, and xylan from birch wood or oat spelt ranged widely. Among the subcloness, p2 and p13 (BAC clones 6-C02 and 14-B22) showed the highest activities towards Avicel and filter paper of the CMCases examined.

**Table 2:** Optimal pH values and temperatures of the cellulases cloned from metagenomic BAC library of the cow rumen

**Cellulase**	**p44**	**p75**	**p13**	**p2**	**p35**	**p44c**	**p61**	**p296**	**p41**	**p28**
Optimal pH	6.5	6.5	6.0	6.5	6.5	6.5	6.5	7.0	7.0	6.5
Optimal temperature (°C)	45	45	50	50	50	50	50	50	40	40

**Table 3:** Substrate specificity of the protein crude extracts from cultures of subclones expressing cellulase activity

**Substrate**	**Relative specific activity (%)**									
	**p44**	**p75**	**p13**	**p2**	**p35**	**p44c**	**p61**	**p296**	**p41**	**p28**
Barley glucan ((1–3,1-4)-β-D-glucan)	100	5.9	15.6	9.2	5.1	20.3	11.1	6.5	2.4	5.3
Lichenan ((1–3,1-4)-β-D-glucan)	80.4	5.6	23.0	5.1	100	28.4	6.0	23.9	1.1	3.3
Carboxymethylcellulose (1,4-β-D-glucan)	53.5	1.2	100	100	50.5	73.0	42.2	78.9	16.8	7.5
Methyl cellulose (1,4-β-D-glucan)	5.3	0.4	12.5	14.2	6.5	8.7	5.8	14.1	0.5	0.3
Avicel (1,4-β-D-glucan)	18.0	0.8	44.8	72.6	21.7	21.1	6.9	62.9	0.7	1.2
Xylan from birchwood (1,4-β-D-xylan)	5.3	0.3	21.3	16.8	6.2	4.9	1.2	18.1	0.2	0.2
Xylan from oat spelt (1,4-β-D-xylan)	12.3	0.6	27.7	18.8	11.4	10.4	5.2	29.8	0.6	1.4
Oat gum ((1–3,1-4)-β-D-glucan)	5.9	100	71.0	14.9	45.5	100	100	100	100	100
Filter paper	4.1	0.2	11.1	7.9	4.6	3.4	1.0	11.8	0.1	0.2

### Overexpression of C6C02 and Cel14b22

**Figure 2:**
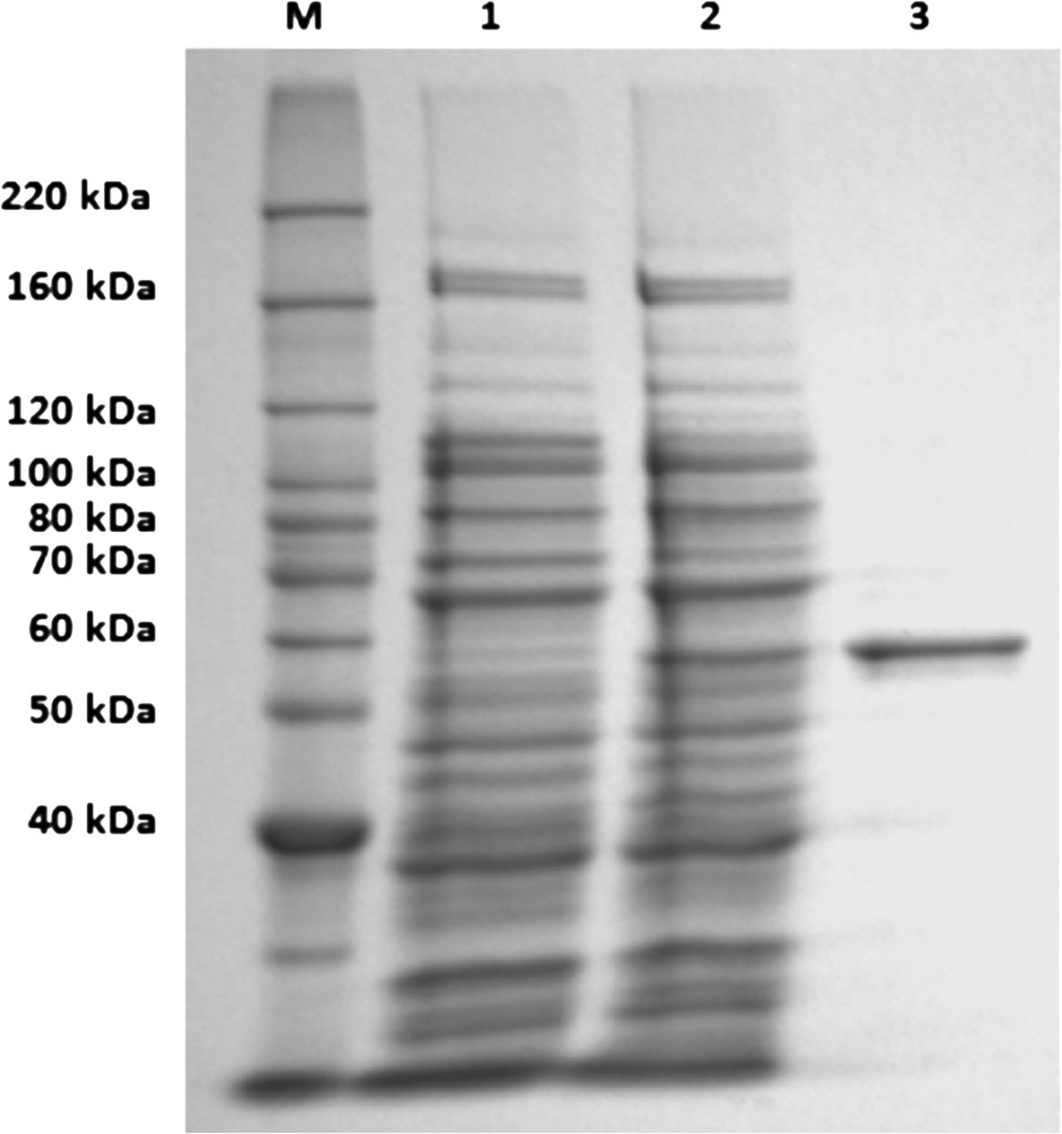
**Sodium dodecyl sulfate polyacrylamide gel electrophoresis (SDS-PAGE) analysis of recombinant Cel14b22 protein stained with Coomassie blue.** Lane M: protein molecular weight marker. Lane 1: Crude extract before IPTG induction. Lane 2: Crude extract after IPTG induction. Lane 3: Purified Cel14b22.

Due to the high activity against crystalline cellulose the cellulases, encoded in subclones p2 and p13 were selected for further study. The genes, designated by the names of the original BAC clones as *C6c02* and *Cel14b22*, had lengths of 1680 bp, encoding for 559 amino acids, and 2679 bp, encoding for 892 amino acids, respectively. SMART analysis of deduced amino acid sequences of the *C6c02* and *Cel14b22* cellulase genes showed that each contained a glycosyl hydrolase (GH) family 5 catalytic domain and a signal peptide. The C6c02 contained a CBM_II, but Cel14b22 contained a C-terminal module with no significant homology to known CBMs. Unfortunately, despite our best efforts the overexpressed product of C6c02 was insoluble and could not be purified. SDS-PAGE analysis of the crude extract of Cel14b22 showed expression of 6xHis tagged proteins, as observed by the appearance of an extra protein band migrating at about 63 kDa upon induction (Figure 2, lane 2). The size of the expressed Cel14b22 was similar to the molecular mass calculated from the amino acid sequences (63 kDa). After purification with the Ni-NTA column and desalting with PD-10 columns, a single band was shown on the SDS-PAGE gel corresponding with the size of the enzyme, suggesting that the enzyme was purified to homogeneity (Figure 2, lane 3).

### Characterization of the purified recombinant Cel14b22

**Figure 3:**
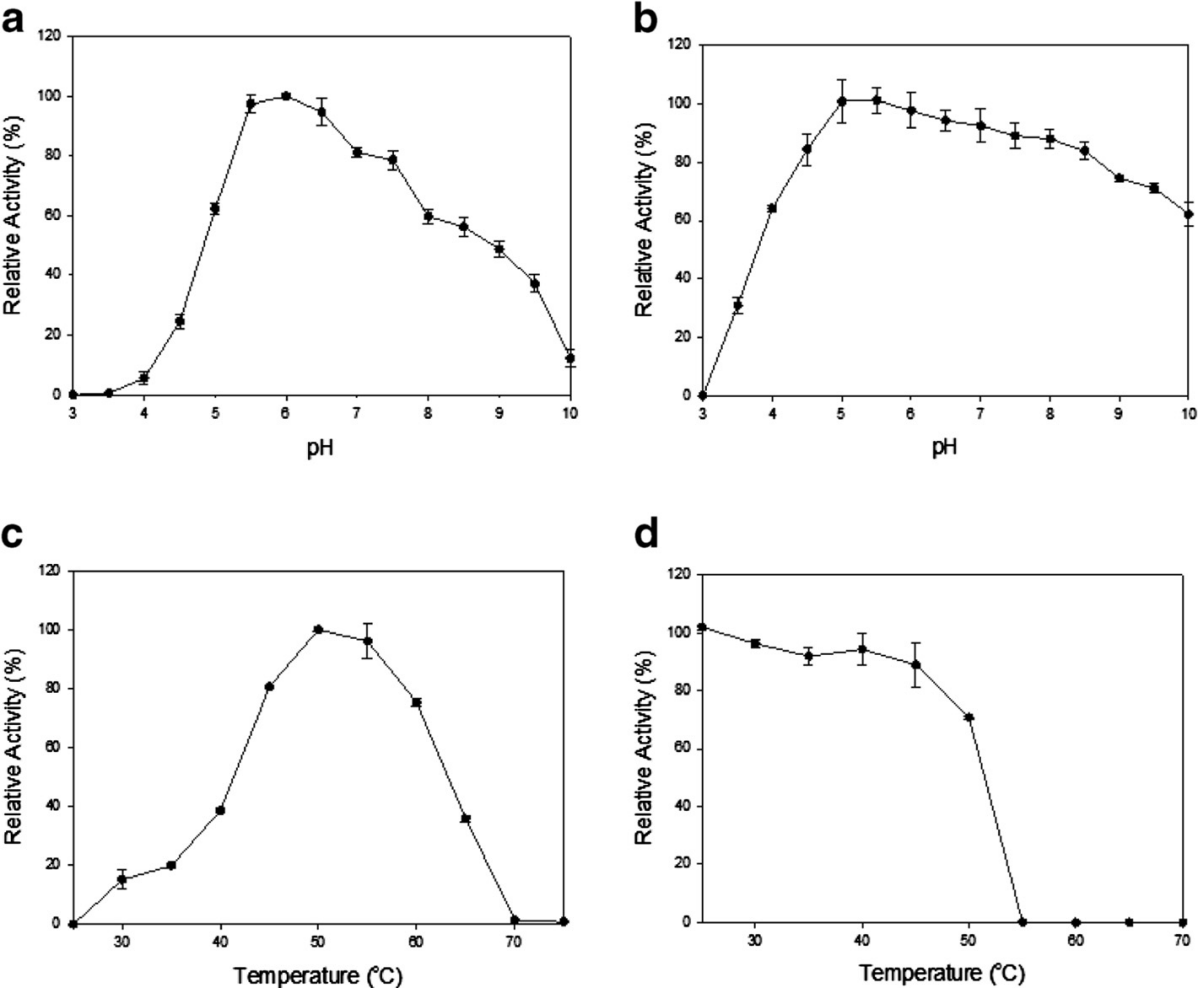
**Effects of pH and temperature on the activity and the stability of Cel14b22. a**) Effect of pH on activity of Cel14b22. **b**) pH stability of Cel14b22. **c**) Effect of temperature on the activity of Cel14b22. **d**) Temperature stability of Cel14b22. The error bars represent the standard deviation of triplicate measurements.

The activity of Cel14b22 towards CMC was optimal at a ~ pH 6.0 and 50°C, consistent with the optimal conditions for the crude protein extract from subclone p13. The Cel14b22 enzyme retained more than 60% of its activity after storage at 4°C for 24 h at pHs ranging from 4 to 10 (Figure 3 a,b). The enzyme was stable for 1 h at temperatures below 50°C with over 80% of the activity remaining, but activity was completely lost at temperatures above 55°C (Figure 3 c,d). The K_*m*_ and V_max_ of the recombinant *Cel14b22* towards CMC were 13.23 mg/mL and 178.57 U/mg, respectively.

Substrate specificity of the recombinant Cel14b22 was determined under optimal conditions with 1% polysaccharides (Table 4). The enzyme had the highest activities towards barley oat gum, and showed low activity toward insoluble celluloses.

**Table 4:** Substrate specificity of Cel14b22

**Substrates**	**Specific activity**^**a **^**(U/mg) ± SD**	**Relative activity (%)**
Oat gum ((1–3,1-4)-β-D-glucan)	368.22 ± 2.77	100
Barley β-glucan ((1–3,1-4)-β-D-glucan)	172.94 ± 9.92	47
Carboxymethyl cellulose (1,4-β-D-glucan)	110.42 ± 4.07	30
Lichenan ((1–3,1-4)-β-D-glucan)	86.48 ± 4.45	23
Oat spelt xylan (1,4-β-D-xylan)	39.79 ± 3.62	11
Methyl cellulose (1,4-β-D-glucan)	39.41 ± 3.72	11
Birchwood xylan (1,4-β-D-xylan)	38.29 ± 5.44	10
Avicel (1,4-β-D-glucan)	21.96 ± 3.40	6
Filter paper	20.78 ± 6.97	6

^a^ The incubation time for activity measurement was 15 min, performed in triplicate and the standard deviations were calculated.

The effects of metal ions, EDTA, and SDS on CMCase activity were also determined. Mn^2+^ enhanced the enzymatic activity to 155%, whereas Cu^2+^ and Fe^3+^ dramatically reduced enzyme activity, to 21% and 12%, respectively. Cr^2+^, Zn^2+^ and Mg^2+^ had only slight inhibitory effects, and Co^2+^, Ca^2+^, K^+^ and Na^+^ did not alter activity. The chelating agent EDTA slightly inhibited activity (to 84%), whereas SDS completely abolished the activity of Cel14b22 (Table 5).

**Table 5:** Effects of metal ions, chelating agent, and detergent on the enzyme activity of Cel14b22

**Reagent**	**Concentration**	**Relative activity (%) ± standard deviation**
Control	-	100
CoCl_2_	10 mM	107.9 ± 4.7
CaCl_2_	10 mM	102.2 ± 5.4
CrCl_2_	10 mM	68.4 ± 1.6
CuSO_4_	10 mM	21.4 ± 0.6
ZnCl_2_	10 mM	61.5 ± 0.7
MgCl_2_	10 mM	85.0 ± 4.6
FeCl_3_	10 mM	12.8 ± 2.3
MnCl_2_	10 mM	155.2 ± 5.4
KCl	10 mM	103.7 ± 6.0
NaCl	10 mM	105.5 ± 4.4
EDTA	1 mM	84.0 ± 4.0
SDS	1% w/v	0

## Discussion

This study was in part undertaken to assess the utility of a freeze grinding approach to the recovery of representative, high molecular weight, metagenomic DNA from the rumen microbial community and to identify cellulases that may be of industrial interest. This approach had a particular focus on the quantitative recovery of DNA from the largely fibre-associated members of the rumen microbial community involved in plant fibre degradation. The test BAC library comprised ~6000 clones constructed with high molecular weight DNA isolated by a freeze grinding technique from dairy cow rumen samples. The total library encompassed an estimated 900 Mb of insertion DNA. The positive rate of hydrolase activity expression in the library was approximately 0.15% of the tested clones, or one expressed hydrolase gene per 10 Mb of insert DNA. For polysaccharidases, the rate was about 1 hydrolase gene per 25 Mb of insert DNA. This rate is very similar to that which would be expected from the data of Brulc et al. [16], who used a pyrosequencing approach, where examination of ~25 000 Mb of sequence data identified ~1000 GH family sequences. This result suggests that the freeze grinding approach indeed provides a representative sample of the rumen metagenome while facilitating a functional screening approach.

We identified twenty five cellulase positive BAC clones (inluding nine β-glucosidases). The high number of hydrolytic clones is consistent with the adaptation of the rumen microbial community for the digestion of plant cell-wall material in the rumen [16,17]. However, cellobiohydrolases or multi-domain cellulases were not retrieved in this study, most likely because that they did fold appropriately in host *E. coli*, were not expressed, or their expression level was too low to be detected. However, pyrosequencing of BAC clones expressing hydrolase activities does allow the identification of clustered genes which might be functionally related to the expressed activity. This is apparent in BAC clone 3-N18, where genes for a xylanase, esterase and 3 putative glycoside hydrolases grouped together. BAC clones that show homologies to *Fibrobacter succinogenes,* a prominent cellulolytic bacterium in the rumen, do not exhibit this pattern, as has been noted in the *F. succinogenes* genome sequence [20].

The recombinant enzyme Cel14b22 was over-expressed and purified. It comprises an N-terminal signal peptide (amino acids 1–19), a catalytic module belonging to the glycoside hydrolase family 5 (amino acids 40–339), and a C-terminal module with no known functional homologue. The conserved catalytic residues were identified by homology to other GH5 family members as the Glu 178 and Glu 289 residues. As with the two catalytic residues, the other six well-established conserved residues in the GH5 family were also verified in the Cel14b22 sequence: Arg 83, His 125, Asn 177, His 253, Tyr 255 and Trp 331. Studies suggested that these eight residues are conserved in all GH5 family enzymes [21,22].

**Figure 4:**
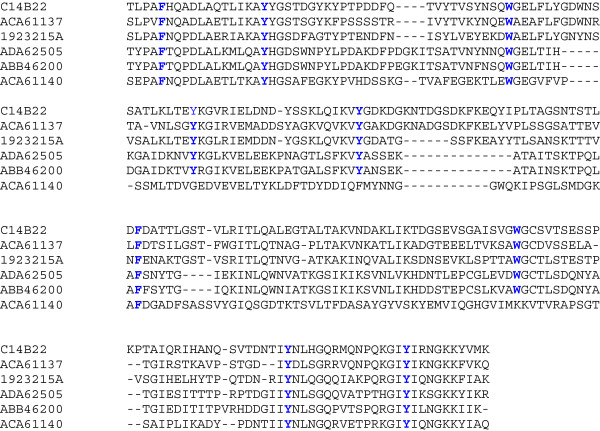
**Multiple sequence alignment of C-terminal module (amino acids 340–559) of Cel14b22 with xylanase/cellulase (1923215A, 375-584aa) from the ruminal bacterium *Prevotella ruminicola *23 and uncultured ruminal microbial cellulases (ACA61137, aa 334–546; ACA61140, aa 334–537; ABB46200, aa 715–917; ADA 62505, aa 721–919).** The nine conserved aromatic amino acid residues among homologous C-terminal modules are highlighted in bold and blue.

The C-terminal module (amino acids 340–559) showed no significant homology to known CBMs, but it shared 28% identity with the C-terminal module of another GH5 cellulase, ACA61140. This GH5 family cellulase, from another uncultured ruminal microorganism, also shows no significant homology to known CBMs [23]. Almost all CBMs studied to date contain surface exposed aromatic rings, which have been shown to be involved in the recognition and binding of polysaccharides. Aromatic amino acid residues form face-to-face hydrophobic stacking interactions in which a tryptophan or tyrosine ring interacts with the nonpolar face of a sugar ring [24]. An alignment of the module with a xylanase/cellulase from the ruminal bacterium *Prevotella ruminicola* 23 (1923215A, aa 375–584), uncultured ruminal microbial cellulases ACA61137 (aa 334–546) and ACA61140 (aa 334-537aa), and uncultured ruminal bifunctional/cellulase enzymes ABB46200 (aa 715–917) and ADA62505 (aa 721–919) showed that nine aromatic amino acid residues were conserved in the sequences (Figure 4) with the two tryptophan residues possibly involved in cellulose binding [25]. The observation that Cel14b22 has activity towards Avicel and filter paper also suggests that the C-terminal module has CBM activity.

Cel14b22 shares physico-chemical properties with many other family 5 endoglucanases. Several cellulases from fungi [26] and bacteria [27,28] show optimal hydrolytic activities under similar conditions. Cel14b22 is active over a wide range of pH, retaining 40% of its activity between pH 4.5 and pH 9.5, and it is stable even at pH 10.0.The Cel14b22 endoglucanase was only partially inhibited when incubated in 1 mM EDTA, suggesting that Cel14b22 does not absolutely require divalent cations to degrade CMC. Furthermore, addition of 10 mm Ca^2+^ or Co^2+^ to the incubation buffer did not alter Cel14b22 activity. This is the case for the majority of glycoside hydrolases from family 5, whereas these cations seem to stimulate several family 9 glycoside hydrolases [29,30]. The metal ion Mn^2+^ did enhance enzyme activity, which is consistent with most cellulases [31-33]. It is known that different metallic ions, such as Fe^3+^, Cu^2+^, Zn^3+^, exert an inhibitory effect on family 5 glycoside hydrolases [30,34], especially those from ruminal cellulolytic bacteria. This was also observed with Cel14b22 with Zn^3+^, Cu^2+^ and Fe^3+^. The activity of Cel14b22 was significantly higher toward mixed-linkage β-glucans from barley and oats than toward (1,4)-β-glucans, including CMC. Activity was also lower toward the mixed-linkage β-glucan lichenan, perhaps due to the higher proportion of β-(1,3)-linkages in this polysaccharide. The Cel14b22 product could also hydrolyse birchwood xylan and oat-spelt xylan, but had limited activity toward microcrystalline cellulose and filter paper, which are largely resistant to hydrolysis by many of the previously characterized GH 5 glucanases [30,35] including the metagenome-derived endoglucanase Cel5A [23]. The broad substrate specificity of Cel14b22 is consistent with previous reports on cellulases from other glucanase families [36-41]. Although the enzyme shows considerable homology (66%) with another uncultured ruminal microbial cellulase [40], its enzymatic characteristics are quite different.

## Conclusions

Our study confirmed the utility of a freeze grinding method for the isolation from a complex microbial community of high molecular weight metagenomic DNA for use in the construction of BAC libraries. Such libraries allow functional screening for the isolation of novel hydrolytic enzymes and facilitate the analysis of gene clusters containing functionally related genes that may or may not be expressed in the heterologous host. In addition we identified and characterized a novel family 5 glycoside hydrolase with significant activity towards a variety of β-1,4- and β-(1,3-1,4) glucans, including microcrystalline and filter paper celluloses, and β-1,4-xylans.

## Methods

### DNA isolation from rumen samples

Rumen contents (mixed solids and liquid) were sampled from a rumen cannulated dairy cow maintained on a grass/hay diet. The samples (~15 g) were centrifuged (19 200 × g, 10 min), and the pellet suspended in 30 mL of 100 mM Tris–HCl pH 8.0, 500 mM EDTA pH 8.0, 1.5 M NaCl, 1 mg/mL Proteinase K and rapidly frozen in liquid nitrogen. Frozen samples were pulverized in a Retsch RM 100 Mortar Grinder (F. Kurt Retsch GmbH and Co., Haan, Germany) and ground in the presence of liquid nitrogen for 5 min. Following grinding, samples were incubated in a water bath at 50°C for 40 min, combined with 3 mL 2% SDS and incubated at 65°C for another 45 min. The lysate was centrifuged at 19 200 × g for 10 min at room temperature to pellet debris and the supernatant was combined 1:1 (v/v) with warm (65°C) 2% agarose (w/v in distilled water) by gentle inversion. This mixture was poured into 90 mm square petri plates to a depth of 5–6 mm thickness and allowed to solidify at room temperature. The agarose containing the embedded DNA was then cut into 5 mm strips and the strips were equilibrated 3 times over 24 h against 30 volumes of TE buffer (10 mM Tris.HCl, 1 mM EDTA, pH 8.0) and stored at 4°C. Low molecular weight DNA (< 25Kb) was removed by a single fractionation by field inversion gel electrophoresis in 1% agarose and 0.5× TBE using a Hoefer PC750 pulse controller (Hoefer Scientific Instruments, San Francisco, CA, USA) set at 3 s forward, 1 s reverse with a 0.5×/h increasing time ramp, run at 4 V.cm-1 for 16 h at 4°C. Fragments of DNA larger than 50 Kb were concentrated by electrophoresis at a constant 4 V/cm onto a piece of 6 000–8 000 Da cutoff dialysis membrane secured in the gel at the 50 Kb point. After sufficient time to move all DNA to the membrane the field was reversed and the DNA run back off the membrane into the gel a distance of 2 mm. The 3 mm strip of agarose containing the concentrated high molecular weight (50 to ~ 500Kb) DNA was removed and stored in sterile TE buffer at 4°C.

### *Sau3A*I digestion and DNA sizing

Agarose containing 50–500 Kb rumen metagenomic DNA was equilibrated three times for 30 min in 1 × New England BioLabs buffer 1, 1 × Bovine Serum Albumin (New England BioLabs, Pickering, ON, Canada). The agarose was then melted at 65°C for 5 min, cooled to 37°C, and *Sau3A*I (New England BioLabs) was added at a concentration of 0.01U/μL of agarose. The mixture was incubated at 37°C for 10 min and digestion stopped by the addition of EDTA to a final concentration of 20 mM, producing a limited partial *Sau3A*I digest. The partially *Sau3A*I-digested DNA was then further sized as described above to remove fragments less than 50 kb before being used for ligation.

### BAC library construction

Agarose containing partially digested and sized metagenomic DNA was equilibrated 3 times over 24 h in 10 volumes of TE. A portion was melted, diluted two-fold and used to determine DNA concentration using a NanoDrop3300 fluorospectrometer (Thermo Scientific NanoDrop, Wilmington DE, USA). Metagenomic DNA (25–50 ng) in no more than 12.5 μL of agarose was used in ligation reactions set up according to the manufacturer’s directions using 25 ng pSMART BAC vector (Lucigen Corporation, Middleton, WI, USA) in a final volume of 50 μL. Ligation reactions (1 μL) were transformed into 20 μL of BAC-Optimized Replicator v2.0 Electrocompetent Cells (Lucigen Corporation) in a 1 mm gap cuvette using a BTX ECM 600 (BTX Harvard Apparatus, Holliston MD, USA) set with a resistance of 129 ohms and charging voltage of 1.2 kV. Positive transformants were selected on YT agar (0.8 g tryptone, 0.5 g yeast extract, 0.5 g NaCl, 1.2 g agar per 100 mL water) containing 5% sucrose and 12.5 μg/mL chloramphenicol. Positive clones were picked using a Genetix QPix (Genetix, San Jose CA, USA) into 384 well plates containing Luria-Bertani (LB) freezing medium (1.0 g tryptone, 0.5 g yeast extract, 1.0 g NaCl, 0.18 g H_2_PO4, 0.67 g K_2_HPO4, 0.05 g sodium citrate, 0.09 g (NH_4_)_2_SO_4_, 4.4 mL glycerol, 20 μL 5 N NaOH, per 100 mL water). After autoclaving sterile Mg SO_4_ was added to a final concentration of 0.4 mM containing 12.5 μg/mL chloramphenicol and clones were cultured overnight at 37°C before storing at −80°C.

### Screening for clones expressing hydrolase activities

Cultures stored in LB freezing medium were replicated into LB with 12.5 μg/mL chloramphenicol in 384 well plates and grown overnight at 37°C. Clones were transferred from the 384 well plates onto 22 cm × 22 cm plates containing LB agar overlaid with a 1 mm deep layer of agar containing the test substrate, in a 3 × 3 macroarray grid pattern using a Genetix QPix, such that each individual clone was inoculated twice in each 3 × 3 grid. A maximum of 6912 clones were screened on each 22 cm × 22 cm plate.

Polysaccharide hydrolase activities were detected using the method of Wood et al. [41] with overlays containing carboxymethyl cellulose (CMC) (Sigma-Aldrich, Oakville, ON, Canada), oat ß glucan, lichenan (Sigma-Aldrich), or corn xylan (Sigma-Aldrich) at a 1% concentration, followed by staining with Congo red to visualize clearing zones. ß-glucosidase activity was evaluated using plates containing 0.1% esculin and 0.05% ferric ammonium citrate in the overlay and observing a black precipitate around clones positive for ß-glucosidase activity [42].

### BAC sequencing and analysis

BAC clones expressing positive cellulase activities were selected for shotgun 454 sequencing (Macrogen, Seoul, Korea). Sequence data was assembled using Mira3 [43] after vector sequences were masked using the program Smalt (Wellcome Trust Sanger Institute, Cambridge, UK). Assembled contigs were imported into Geneious [44] (Biomatters Ltd., Auckland, New Zealand) and open reading frames were identified using open reading frame finder (NCBI; http://www.ncbi.nlm.nih.gov) and Glimmer [45] Sequences identities were investigated using BlastN, BlastX and BlastP (NCBI; http://www.ncbi.nlm.nih.gov) and genome comparisons were made using IMG and IMG/M [46]. The modular structures of the enzymes were predicted by SMART online ( http://smart.embl-heidelberg.de). The nucleotide sequence of the cellulase gene (Cel14b22) was deposited into the GenBank database under accession number JN98181.

### Subcloning and characterization of cellulase activities

Subcloning was performed to localize the hydrolase genes, to shorten the inserts for effective sequencing, and to increase expression levels for biochemical characterization. Extracted BAC DNA was obtained from hydrolase-positive clones using a large and partial digest with *Sau3A*I. Fragments between 2 and 8 kb were extracted from agarose gel with a QIAquick Gel Extraction Kit (Qiagen, Mississauga, ON, Canada) according to the manufacturer directions and subcloned into *BamH*I digested, calf intestinal phosphatase dephosphorylated plasmid vector pUC19 (Invitrogen, Burlington, ON, Canada). The resulting transformants were screened for cellulase activity as described above. Ten subclones expressing different CMC hydrolases (CMCases) were identified and grown in 200-mL cultures overnight. The cells were harvested by centrifugation (8000 × g, 15 min), resuspended in 4 mL of 0.1 M sodium-phosphate buffer (pH 6.5) and lysed by sonication. Crude cell lysates were centrifuged at 10 000 × g for 20–30 min at 4°C to remove cellular debris and supernatants were used as crude protein extracts for subsequent assays. pH profiles for individual subclones was determined at 40°C by monitoring enzyme activity over a range of pHs (0.1 M citric acid-sodium citrate buffer, pH 3.0~6.0; 0.1 M sodium-phosphate buffer, pH 6.0~8.0; 0.1 M glycine-NaOH buffer, pH 8.6~10.0). Optimal activity over a temperature range of 25°C to 75°C was also determined. To analyse substrate specificities, the hydrolase activity of the protein extracts was measured after 30 min incubation at the optimal temperature in the optimal buffer containing 1% (w/v) polysaccharides. The tested polysaccharides were CMC, Avicel, lichenan, barley glucan, methyl cellulose, oat spelt xylan, birch wood xylan, oat gum, and filter paper. Reducing sugars released from the substrates were measured with 3,5-dinitrosalicylic acid as described by Miller [47]. One unit (U) of endoglucanase activity was defined as the amount of enzyme releasing 1 μmol of reducing sugar per min from the substrate. Equal amounts of protein were added to each reaction and the highest enzymatic activity towards a substrate for each crude enzyme extract from each subclone was used as benchmark of 100% activity.

### SDS-PAGE gel electrophoresis, native PAGE and hydrolase zymography

Sodium dodecyl sulfate polyacrylamide gel electrophoresis (SDS-PAGE) was performed according to the method of Laemmli [48]. Native PAGE was carried out similarly with the exclusion of SDS from all solutions. For zymogram analysis of endoglucanases, the gel was placed on a fresh 1% agar plate containing 0.2% (w/v) CMC, and the covered plate was incubated at 37°C for 1 h followed by staining and destaining with Congo Red as described above for library screening.

### Expression and purification of recombinant enzyme Cel14b22

The endoglucanase gene harboured in BAC clone 14b22 (subclone p13), designated *Cel14b22*, was amplified by polymerase chain reaction (PCR) with the primers 5' GGAAGATCTTATGAAGAAAATTCTACT -3' (forward) and 5'- CCGGAATTCTTATTTCATAACGTATT -3' (reverse). PCR product was digested with *Bgl*II and *EcoR*I and ligated into the expression vector pET-30a (+) for expression of the recombinant protein with an N-terminal 6-His tag. The resulting expression construct was transformed into *Escherichia coli* BL21(DE3) and positive clones were selected on plates containing kanamycin. Cells were grown in LB broth containing 25 mg/mL of kanamycin at 37°C with shaking at 200 rpm and protein expression was induced with 1 mM IPTG (final concentration) when an optical density (600 nm) of 0.6 was reached. Protein expression was carried out at room temperature for 3–5 h. Cells were harvested by centrifugation (5000 × g, 15 min), resuspended in lysis buffer (0.05 M NaH_2_PO_4_; pH 8.0, 0.300 M NaCl, 0.02 M imidazole) and lysed by sonication. Cell debris were pelleted by centrifugation (30 000 × g for 30 min) and the supernatant was loaded onto a 2 mL Ni-NTA (nickel-nitrilotriacetic acid) column. The imidazole concentration was increased to 0.04 M and the resin was washed with 10 mL of buffer. Protein was eluted by increasing the imidazole to 0.25 M in the final wash. The final purified protein solution was desalted using a PD-10 ultrafiltration column (GE Healthcare, Mississauga, ON, Canada) by gravity flow according to the manufacturer’s directions and eluted with 0.05 M citrate-phosphate buffer, pH 6.0. Protein concentration was determined using a Quick Start Bradford Protein Assay kit (Bio-Rad, Mississauga, ON, Canada).

### Characterization of Cel14b22 activities

Cellulase activity was measured by incubating 0.7 μg of recombinant Cel14b22 with 1% CMC in 0.5 mL 0.1 M citrate phosphate buffer, pH 6.0, at 50°C for 15 min. Optimal pH and temperature were determined as described above. Substrate specificities of the enzyme and the effect of several metal chloride salts at 10 mM (Ca, Co, Cr, Cu, Fe, K, Mg, Mn, Na, Zn), a chelating agent (EDTA) at 1 mM, and the detergent SDS at 1% (w/v) were investigated at optimal pH (6.0) and temperature (50°C). The pH stability was determined by measuring residual cellulase activity after the enzyme was incubated at 4°C for 24 h at the test pH. Thermal stability data were compared after incubating the enzyme at various temperatures from 30 to 70°C for 1 h, and measuring the residual cellulase activity. The kinetic constants, K_m_ and V_max_, were calculated by directly fitting the data to the Michaelis–Menten equation by nonlinear regression. Reactions were carried out under optimal condition with CMC of different concentrations, ranging from 5 to 35 mg/mL.

## Abbreviations

BAC: Bacterial artificial chromosome; CBM: Carbohydrate binding module; CMC: Carboxymethylcellulose; EDTA: Ethylenediaminetetraacetic acid; GH: Glycosyl hydrolase; SDS-PAGE: Sodium dodecyl sulfate polyacrylamide gel electrophoresis; Ni-NTA: Nickel-nitriloacetic acid.

## Competing interests

The authors declare no competing interests.

## Authors’ contribution

XG carried out functional screening and characterized the enzymes. LP isolated metagenomic DNA and constructed the BAC library. RG and SQ analysed data and wrote the manuscript. RF, TM, RT conceived of the study and participated in its design and coordination and helped to draft the manuscript. All authors read and approved the final manuscript.

## References

[B1] Lynd LR van Zyl WH McBride JE Laser M Consolidated bioprocessing of cellulosic biomass: an update Curr Opin Biotechnol 2005 16 557 583 10.1016/j.copbio.2005.08.009 16154338

[B2] Bhat MK Cellulases and related enzymes in biotechnology Biotechnol Adv 2000 18 355 383 10.1016/S0734-9750(00)00041-0 14538100

[B3] Kristensen JB Thygesen LG Felby C Jørgensen H Elder T Cell-wall structural changes in wheat straw pretreated for bioethanol production Biotechnol Biofuels 2008 1 5 13 10.1186/1754-6834-1-5 18471316 PMC2375870

[B4] Russell JB Rychlik JL Factors that alter rumen microbial ecology Science 2001 292 1119 1122 10.1126/science.1058830 11352069

[B5] Krause DO Denman SE Mackie RI Morrison M Rae AL Attwood GT McSweeney CS Opportunities to improve fiber degradation in the rumen: microbiology ecology and genomics FEMS Microbiol Rev 2003 27 663 693 10.1016/S0168-6445(03)00072-X 14638418

[B6] Pace NR A molecular view of microbial diversity and the biosphere Science 1997 276 734 740 10.1126/science.276.5313.734 9115194

[B7] Rondon MR Goodman RM Handelsman J The Earth’s bounty: assessing and accessing soil microbial diversity Trends Biotechnol 1999 17 403 409 10.1016/S0167-7799(99)01352-9 10481172

[B8] Kocherginskaya SA Aminov RI White BA Analysis of the rumen bacterial diversity under two different diet conditions using denaturing gradient gel electrophoresis random sequencing and statistical ecology approaches Anaerobe 2001 7 119 134 10.1006/anae.2001.0378

[B9] Galbraith EA Antonopoulos DA White BA Suppressive subtractive hybridization as a tool for identifying genetic diversity in an environmental metagenome: the rumen as a model Environ Microbiol 2004 6 928 937 10.1111/j.1462-2920.2004.00575.x 15305918

[B10] Edwards E McEwan R Travis A Wallance R 16S rDNA library-based analysis of ruminal bacterial diversity Antonie Van Leeuwenhoek 2004 86 263 281 15539930 10.1023/B:ANTO.0000047942.69033.24

[B11] Shin EC Cho KM Lim WJ Hong SY An CL Kim EJ Kim YK Choi BR An JM Kang JM Kim H Yun HD Phylogenetic analysis of protozoa in the rumen contents of cow based on the 18S rDNA sequences J Appl Microbiol 2004 97 378 383 10.1111/j.1365-2672.2004.02304.x 15239705

[B12] Ohene-Adjei S Chaves AV McAllister TA Benchaar C Teather RM Forster RJ Evidence of increased diversity of methanogenic archaea with plant extract supplementation Microb Ecol 2008 56 234 242 10.1007/s00248-007-9340-0 18075710

[B13] Tringe SG von Mering C Kobayashi A Salamov AA Chen K Chang HW Podar M Short JM Mathur EJ Detter JC Bork P Hugenholtz P Rubin EM Comparative metagenomics of microbial communities Science 2005 308 554 557 10.1126/science.1107851 15845853

[B14] Ferrer M Golyshina OV Chernikova TN Khachane AN Reyes-Duarte D Santos VA Strompl C Elborough K Jarvis G Neef A Yakimov MM Timmis KN Golyshin PN Novel hydrolase diversity retrieved from a metagenome library of bovine rumen microflora Environ Microbiol 2005 7 1996 2010 10.1111/j.1462-2920.2005.00920.x 16309396

[B15] Lorenz P Eck J Metagenomics and industrial applications Nat Rev Microbiol 2005 3 510 516 10.1038/nrmicro1161 15931168

[B16] Brulc JM Antonopoulos DA Miller ME Wilson MK Yannarell AC Dinsdale EA Edwards RE Frank ED Emerson JB Wacklin P Coutinho PM Henrissat B Nelson KE White BA Gene-centric metagenomics of the fiber-adherent bovine rumen microbiome reveals forage specific glycoside hydrolases Proc Natl Acad Sci USA 2009 106 1948 1953 10.1073/pnas.0806191105 19181843 PMC2633212

[B17] Hess M Sczyrba A Egan R Kim TW Chokhawala H Schroth G Luo S Clark DS Chen F Zhang T Mackie RI Pennacchio LA Tringe SG Visel A Woyke T Wang Z Rubin EM Metagenomic discovery of biomass-degrading genes and genomes from cow rumen Science 2011 331 463 467 10.1126/science.1200387 21273488

[B18] Warnecke F Luginbühl P Ivanova N Ghassemian M Richardson TH Stege JT Cayouette M McHardy AC Djordjevic G Aboushadi N Sorek R Tringe SG Podar M Martin HG Kunin V Dalevi D Madejska J Kirton E Platt D Szeto E Salamov A Barry K Mikhailova N Kyrpides NC Matson EG Ottesen EA Zhang X Hernández M Murillo C Acosta LG Rigoutsos I Tamayo G Green BD Chang C Rubin EM Mathur EJ Robertson DE Hugenholtz P Leadbetter JR Metagenomic and functional analysis of hindgut microbiota of a wood-feeding higher termite Nature 2007 450 560 565 10.1038/nature06269 18033299

[B19] Zhu L Wu Q Dai J Zhang S Wei F Evidence of cellulose metabolism by the giant panda gut microbiome Proc Natl Acad Sci USA 2011 108 17714 177149 10.1073/pnas.1017956108 22006317 PMC3203778

[B20] Suen G Weimer PJ Stevenson DM Aylward FO Boyum J Deneke J Drinkwater C Ivanova NN Mikhailova N Chertkov O Goodwin LA Currie CR Mead D Brumm PJ The complete genome sequence of Fibrobacter succinogenes S85 reveals a cellulolytic and metabolic specialist PLoS One 2011 6 1 15 10.1371/journal.pone.0018814 PMC3079729 21526192

[B21] Sakon J Adney WS Himmel ME Thomas SR Karplus PA Crystal structure of thermostable family 5 endocellulase E1 from Acidothermus cellulolyticus in complex with cellotetraose Biochemistry 1996 35 10648 10660 10.1021/bi9604439 8718854

[B22] Lo Leggio L Larsen S The 1.62 Ǻ structure of Thermoascus aurantiacus endoglucanase: completing the structural picture of subfamilies in glycoside hydrolase family 5 FEBS Lett 2002 523 103 108 10.1016/S0014-5793(02)02954-X 12123813

[B23] Duan CJ Liu JL Wu X Tang JL Feng JX A novel carbohydrate-binding module identified in a ruminal metagenomic endoglucanase Appl Environ Microbiol 2010 76 4867 4870 10.1128/AEM.00011-10 20472722 PMC2901718

[B24] Simpson PJ Xie HF Bolam DN Gilbert HJ Williamson MP The structural basis for the ligand specificity of family 2 carbohydrate-binding modules J Biol Chem 2000 275 41137 41142 10.1074/jbc.M006948200 10973978

[B25] Linder M Teeri TT The roles and function of cellulose-binding domains J Biotechnol 1997 57 15 28 10.1016/S0168-1656(97)00087-4 9335165

[B26] Macarron R Acebal C Castillon MP Dominguez JM de la Mata I Pettersson G Tomme P Claeyssens M Mode of action of endoglucanase III from Trichoderma reesei Biochem J 1993 289 867 873 8435082 10.1042/bj2890867 PMC1132256

[B27] Cavicchioli R Watson K Molecular cloning expression and characterization of endoglucanase genes from fibrobacter succinogenes ARI Appl Environ Microbiol 1991 57 359 365 2014986 10.1128/aem.57.2.359-365.1991 PMC182718

[B28] Sheweita SA Ichi-Ishi A Park JS Liu C Malburg LM Doi RH Characterization of engF a gene for a non-cellulosomal Clostridium cellulovorans endoglucanase Gene 1996 182 163 167 10.1016/S0378-1119(96)00544-6 8982083

[B29] Chauvaux S Beguin P Aubert JP Bhat KM Gow LA Wood TM Bairoch A Calcium-binding affinity and calcium-enhanced activity of Clostridium thermocellum endoglucanase D Biochem J 1990 265 261 265 2302168 10.1042/bj2650261 PMC1136638

[B30] Béra-Maillet C Arthaud L Abad P Rosso MN Biochemical characterization of MI-ENG1 a family 5 endoglucanase secreted by the root-knot nematode Meloidogyne incognita Eur J Biochem 2000 267 3255 3263 10.1046/j.1432-1327.2000.01356.x 10824111

[B31] Voget S Steele HL Streit WR Characterization of a metagenome-derived halotolerant cellulose J Biotechnol 2006 126 26 36 10.1016/j.jbiotec.2006.02.011 16584799

[B32] Feng Y Duan CJ Pang H Mo XC Wu CF Yu Y Hu YL Wei J Tang JL Feng JX Cloning and identification of novel cellulase genes from uncultured microorganisms in rabbit cecum and characterization of the expressed cellulases Appl Microbiol Biotechnol 2007 75 319 328 10.1007/s00253-006-0820-9 17216439

[B33] Pang H Zhang P Duan CJ Mo X Ch Tang JL Feng JX Identification of cellulase genes from the metagenomes of compost soils and functional characterization of one novel endoglucanase Curr Microbiol 2009 58 404 408 10.1007/s00284-008-9346-y 19159974

[B34] Rubini MR Dillon AJP Kyaw CM Faria FP Poças-Fonseca MJ Silva-Pereira I Cloning characterization and heterologous expression of the first Penicillium echinulatum cellulase gene J Appl Microbiol 2009 108 1187 1198 19793137 10.1111/j.1365-2672.2009.04528.x

[B35] Fontes CM Clarke JH Hazlewood GP Fernandes TH Gilbert HJ Ferreira LM Possible roles for a non-modular thermostable and proteinase-resistant cellulase from the mesophilic aerobic soil bacterium Cellvibrio mixtus Appl Microbiol Biotechnol 1997 48 473 479 10.1007/s002530051082 9390455

[B36] Wang WY Reid SJ Thomson JA Transcriptional regulation of an endoglucanase and a cellodextrinase gene in Ruminococcus flavefaciens J Gen Microbiol 1993 139 1219 1226 10.1099/00221287-139-6-1219 8360615

[B37] Han SJ Yoo YJ Kang HS Characterization of a bifunctional cellulase and its structural gene the cell gene of Bacillus sp D04 has exo- and endoglucanase activity. J Biol Chem 1995 270 26012 26019 10.1074/jbc.270.43.26012 7592793

[B38] Cho KM Hong SY Lee SM Kim YH Kahng GG Kim H Yun HD A cel44C-man26A gene of endophytic Paenibacillus polymyxa GS01 has multi-glycosyl hydrolases in two catalytic domains Appl Microbiol Biotechnol 2006 73 618 630 10.1007/s00253-006-0523-2 16912849

[B39] Kim YG Choi G Kim S Yoon G Kim Y Ryu Y Screening and characterization of a novel esterase from a metagenomic library Protein Expr Purif 2006 45 315 323 10.1016/j.pep.2005.06.008 16061395

[B40] Duan CJ Xian L Zhao GC Feng Y Pang H Bai JL Tang QS Feng JX Isolation and partial characterization of novel genes encoding acidic cellulases from metagenomes of buffalo rumens J Appl Microbiol 2009 107 245 256 10.1111/j.1365-2672.2009.04202.x 19302301

[B41] Wood PJ Erfle JD Teather RM Use of complex formation between congo red and polysaccharides in the detection and assay of polysaccharide hydrolases Meth Enzymol 1988 160 59 74

[B42] Eberhart B Cross DF Chase LR β-Glucosidase system of Neurospora crassa I β-Glucosidase and cellulase activities of mutant and wild-type strains J Bacteriol 1964 87 761 770 14137612 10.1128/jb.87.4.761-770.1964 PMC277090

[B43] Chevreux B Wetter T Suhai S Genome sequence assembly using trace signals and additional sequence information Comput Sci Biol 1999 99 45 56

[B44] Drummond AJ Ashton B Buxton S Cheung M Cooper A Duran C Field M Heled J Kearse M Markowitz S Moir R Stones-Havas S Sturrock S Thierer T Wilson A Geneious v5 4 [ http://www.geneious.com/]

[B45] Delcher AL Bratke KA Powers EC Salzberg SL Identifying bacterial genes and endosymbiont DNA with Glimmer Bioinformatics 2007 15 673 679 17237039 10.1093/bioinformatics/btm009 PMC2387122

[B46] Markowitz VM Ivanova NN Szeto E Palaniappan K Chu K Dalevi D Chen IM Grechkin Y Dubchak I Anderson I Lykidis A Mavromatis K Hugenholtz P Kyrpides NC IMG/M: a data management and analysis system for metagenomes Nucleic Acids Res 2008 36 534 538 10.1093/nar/gkm869 PMC2238950 17932063

[B47] Miller GL Use of dinitrosalicylic acid reagent for determination of reducing sugar Anal Chem 1959 31 426 428 10.1021/ac60147a030

[B48] Laemmli UK Cleavage of structural proteins during the assembly of the head of bacteriophage T4 Nature 1970 227 680 685 10.1038/227680a0 5432063

